# Pictionary-based fMRI paradigm to study the neural correlates of spontaneous improvisation and figural creativity

**DOI:** 10.1038/srep10894

**Published:** 2015-05-28

**Authors:** Manish Saggar, Eve-Marie Quintin, Eliza Kienitz, Nicholas T. Bott, Zhaochun Sun, Wei-Chen Hong, Yin-hsuan Chien, Ning Liu, Robert F. Dougherty, Adam Royalty, Grace Hawthorne, Allan L. Reiss

**Affiliations:** 1 Center for Interdisciplinary Brain Sciences Research, Department of Psychiatry and Behavioral Sciences, Stanford University School of Medicine, 401 Quarry Road, Stanford, CA 94305; 2 Pacific Graduate School of Psychology-Stanford University Psy.D. Consortium, 1791 Arastradero Road, Palo Alto, CA 94304; 3 Institute of Biomedical Engineering, National Taiwan University, No. 1, Sec. 4, Roosevelt Road, Taipei, 10617, Taiwan; 4 Taipei City Hospital, Zhong-Xing Branch, No. 145, Datong Rd, 10341, Taipei, Taiwan; 5 Center for Cognitive and Neurobiological Imaging, Stanford University, 450 Serra Mall, Building 420, Stanford, CA 94305; 6 Hasso Plattner Institute of Design, Stanford University, Building 550, 416 Escondido Mall, Stanford, CA 94305; 7 Brain and Language Lab, School of English for International Business, Guangdong University of Foreign Studies, Guangzhou, 510420.China; 8 Department of Radiology, Stanford University School of Medicine, 300 Pasteur Road, Stanford, CA 94305

## Abstract

A novel game-like and creativity-conducive fMRI paradigm is developed to assess the neural correlates of spontaneous improvisation and figural creativity in healthy adults. Participants were engaged in the word-guessing game of Pictionary^TM^, using an MR-safe drawing tablet and no explicit instructions to be “creative”. Using the primary contrast of drawing a given word versus drawing a control word (zigzag), we observed increased engagement of cerebellum, thalamus, left parietal cortex, right superior frontal, left prefrontal and paracingulate/cingulate regions, such that activation in the cingulate and left prefrontal cortices negatively influenced task performance. Further, using parametric fMRI analysis, increasing subjective difficulty ratings for drawing the word engaged higher activations in the left pre-frontal cortices, whereas higher expert-rated creative content in the drawings was associated with increased engagement of bilateral cerebellum. Altogether, our data suggest that cerebral-cerebellar interaction underlying implicit processing of mental representations has a facilitative effect on spontaneous improvisation and figural creativity.

Creativity – the ability to create novel but appropriate outcomes, is considered as the driving force behind all human progress. Given the wide import of creativity and its association with mental health across the life span^1^,^2^, it is quintessential to examine the neural networks associated with creative thinking so that novel interventions to foster creativity can be developed. Previously several neuroimaging studies of creativity have been conducted. However, these studies have produced varied findings^3^, with little overlap^4^. Methodological issues might account for this variation, particularly, the inherent elusiveness of the creativity construct itself, diversity in assessments, and the wide range of experimental procedures currently employed^5^,^6^.

Recent neuroimaging studies have devised new avenues for exploring the neural basis of applied creativity. For example, by comparing functional brain activation in artists with non-artists, researchers examined the neural correlates of enhanced artistic creativity^7^,^8^,^9^. Similarly, the neural correlates of musical improvisation have been examined to better understand the brain processes that give rise to enhanced extemporaneity and creativity in musicians^10^,^11^,^12^,^13^,^14^,^15^. Additionally, the brain basis of specific components of creativity, e.g., the “Aha! moment“^16^ and visual creativity^17^, have also been recently examined.

Despite this recent progress, experimental paradigms that are both conducive to creative thinking and facilitate examination of applied creativity remain scarce. Such paradigms could play an essential role in reducing variation in creativity neuroimaging results by minimizing confounding influences of cognitive processes that might not be related to creative thinking but are employed, in part, due to the task design. For example, administering creativity assessments in a test-like setting as opposed to a fun/game-like style can negatively influence creativity^3^,^18^. However, most previous neuroimaging studies of creativity have used traditional test-like assessments. Similarly, performance anxiety can negatively impact creativity^19^, thereby potentially leading to methodological confounds when researchers explicitly ask participants to be “creative”. Lastly, few neuroimaging paradigms allow participants to express their creative potential in a direct/unrestricted manner, as opposed to pressing buttons or “thinking” creatively.

To address some of these issues, we present a novel game-like and creativity-conducive fMRI paradigm to assess the neural correlates of spontaneous improvisation and figural creativity. Here, participants played the word-guessing game of Pictionary^TM^, using an MR-safe drawing tablet, and drew representations of a given word in 30s with a caveat that others would later guess the word by their drawing alone (Fig. 1). The drawings were later scored for creative content and subjective ease of guessing by two experts. Thus, with no explicit instructions to be “creative”, our game-like paradigm was designed to putatively reveal the neural correlates of spontaneous improvisation and applied creativity in healthy adults.

## Results

### Behavioral Results on the fMRI task

The mean rating scores for representation and creativity (on a scale of 1 to 5), across participants, were 3.56 (SD = 0.39) and 2.69 (SD = 0.25), while the mean self-reported difficulty rating (on a scale of 1 to 3) score was 1.83 (SD = 0.25). The representation and creativity rating scores were positively correlated (r(30) = 0.71, p < 0.001), indicating that drawings that were good representations of the given word were also creative. No other significant correlation was observed between difficulty ratings and representation or creativity ratings (*p*′s > 0.05).

### Neural correlates of spontaneous improvisation and figural creativity

We hypothesized that by contrasting word-drawing blocks with control zigzag-drawing blocks we could reveal the neural correlates of spontaneous improvisation and creativity. Using this contrast, increased activation was observed in six different clusters with peak cluster activations bilaterally in the areas of paracingulate gyrus, middle frontal gyrus, superior frontal gyrus, precentral gyrus, thalamus, cerebellum, left lateralized in the occipital cortex, superior parietal lobule, precuneus, and right lateralized in the inferior frontal gyrus (pars triangularis). The cluster with peak activation in the paracingulate gyrus, also extended to the regions of anterior cingulate cortex (ACC), left dorsolateral prefrontal cortex (DLPFC), and left frontal operculum/anterior insula complex (fO-AI). Figure 2 shows the activation map for the primary contrast (in red-yellow color scale) and Table 1A provides information pertaining to the number of voxels and cluster-corrected p-values for each cluster.

For the reverse contrast, i.e., comparing zigzag-drawing with word-drawing condition, we observed widespread activation in the medial-prefrontal cortices, posterior cingulate/precuneus cortex, inferior parietal lobule, lingual gyrus, temporal pole, middle/superior temporal gyrus (posterior division), inferior temporal gyrus (anterior division), postcentral gyrus, paracingulate gyrus, parahippocampal gyrus (posterior division), central/parietal opercular cortex, planum polare, heschl’s gyrus, and planum temporale (Fig. 2 and Table 1B). This result, consistent with known activation of resting-state networks^20^,^21^,^22^, is not surprising given the fact that, compared to word-drawing, zigzag-drawing required minimal cognitive effort from the participants.

To examine how differential activation from the primary contrast (i.e., word- versus zigzag-drawing) is associated with fMRI task performance, we examined the relations between beta-estimates from all the six clusters and behavioral measures of task performance. We observed a significant negative relation between the percentage beta values extracted from the cluster with a peak activation in the paracingulate gyrus (with activations extending into ACC/DLPFC) and representation rating scores (r(30) = −0.430 (95% CI: −0.6843 to −0.0825), p = 0.018; Fig. 2B). This negative relation suggests that higher engagement of the paracingulate (and ACC/DLPFC) regions could potentially lead to lower performance on the fMRI task of word-drawing.

### Parametric modulation of brain activity pattern using rating scores

For each presented word, we obtained a self-reported subjective difficulty rating score from the participants at a post-scan session. As noted above, experts also rated each drawing on task performance (representation and creativity). By parametrically modulating fMRI activation during the word-drawing condition with these three rating scores (in a multiple regression with the two task conditions also included), we identified brain regions that are uniquely and increasingly recruited with corresponding increases in subjective difficulty in word-drawing, and expert ratings of representation and creativity (Fig. 3le 1C). Increased recruitment of the left lateralized middle frontal gyrus, frontal pole, precentral gyrus, and DLPFC was observed in association with increasing subjective difficulty in word-drawing. Increased recruitment in the bilateral cerebellum, lingual gyrus, brain stem, left occipital fusiform, right temporal fusiform, and right inferior temporal gyrus was observed with increasing creativity ratings. No significant results were observed with increasing representation scores. In summary, the left prefrontal regions were increasingly recruited as subjective difficulty increased, while the bilateral cerebellum and inferior temporal gyrus was increasingly recruited in association with more highly rated creative drawings.

## Discussion

We present a novel game-like and creativity-conducive fMRI paradigm to assess the neural correlates of spontaneous improvisation and figural creativity in healthy adults. We strived to keep the environment conducive to intuitive creative thinking by providing an MR-safe drawing tablet and no explicit instructions to be “creative” during the word-guessing game of Pictionary^TM^. The primary contrast of word- versus zigzag-drawing revealed increased engagement of the cerebellum, thalamus, left parietal cortex, right superior frontal, left prefrontal and paracingulate/cingulate regions. Further, higher activation in the cingulate and prefrontal regions was linked to lower expert representation rating scores. The parametric fMRI approach revealed increasing subjective word-drawing difficulty was associated with increasing activation in the left pre-frontal cortices, whereas increasing expert creativity rating was associated with higher activation of the bilateral cerebellum.

Since J. P. Guilford’s seminal lecture on the need to study creativity^23^, a wide range of behavioral and neuroimaging studies have been undertaken to better understand creativity. Unfortunately, the extant literature does not provide converging evidence in terms of specific brain regions/networks that are associated with and/or engaged during creative thinking^3^,^4^. Apart from the methodological issues (e.g., varied experimental designs), such lack of convergence could also be due to several theoretical factors. For example, it has been argued that treating creativity as a monolithic entity is one reason for such variegated findings^24^. Creative thinking, like any other thought process, undoubtedly requires a multitude of explicit brain processes and networks to define the problem/opportunity at hand, to ideate and evaluate different solutions, and to prototype solutions in an iterative fashion. Further, each of these brain processes can in turn be facilitated and adapted to a given problem/situation as a result of the implicit brain processing (e.g., cerebellar facilitation of mental representation manipulations^25^). Thus, moving forward, it is essential to assess creative thinking in terms of neural models of brain processes and their associated interactions. Such neural models would also provide a framework for generating testable hypotheses and for making valid inferences from neuroimaging studies of creativity, with the goal of moving the field of creativity neuroscience towards convergence.

As a starting point, we propose to adapt the neural model proposed by Ito (2008), which includes both explicit and implicit processes potentially engaged during a problem-solving thought^25^. The explicit processes include (a) the working-memory system (to retain information regarding the problem and its constraints within a mentally “graspable” range^26^,^27^); (b) the two task-control attentional systems (adaptive system and stable goal-directed system)^28^; (c) a novelty system to evaluate whether each tentative solution is novel or not^29^; and (d) a system to store mental models and representations, on which all other systems perform actions. The implicit processes, on the other hand, include cerebral-cerebellar interactions to create inverse and forward models that facilitate and increase efficiency of repetitive actions on mental representations. These implicit processes are thought to enhance the likelihood of more creative solutions^25^,^26^. Previous theoretical papers have suggested extending Ito’s and related models for understanding creative thinking^25^,^26^. For example, when using divergent thinking tasks to assess creative capacity, the model predicts explicit systems (especially, adaptive attentional and novelty systems) to be highly activated as the participants are explicitly trying to generate alternative, novel and unique solutions to an open-ended problem. Similarly, for an ‘intuitive leap’ or Aha! moment to happen, the model predicts use of implicit processing (via inverse/forward modeling), where the leap occurs when the solution reaches conscious awareness.

Using Ito’s model as a framework, here we did not expect significant engagement of the novelty system because the participants were not explicitly asked to create novel solutions. Further, as the participants were asked to spontaneously improvise drawings for a given verb/action, we expected increased engagement of regions implicated in implicit processing for both efficient manipulation of mental representations and enhanced creative content in the drawings. As expected, by contrasting word-drawing with zigzag-drawing, we did not observe differential recruitment in the well described novelty system (consisting of hippocampal regions and ventral tegmental area^30^). However, increased engagement of the paracingulate cortex, dorsal ACC, left DLPFC, and left fO-AI complex during the word-drawing condition as compared to zigzag-drawing was observed. Activation in the left fronto-parietal regions suggest involvement of the central executive and “visual sketchpad” of the working memory system^31^,^32^. Further, engagement of the DLPFC, dorsal ACC, fO-AI complex, superior parietal lobule and thalamus suggest activation of both fronto-parietal and cingulo-opercular components of task-control attentional systems during the word-drawing condition. The fronto-parietal component has been proposed to initiate and adjust control on a trial-by-trial basis, whereas the cingulo-opercular component provides stable ‘goal-maintenance’ over the entire task^33^.

In a recent study, and the only other to use an MR-safe drawing tablet, Ellamil and colleagues (2012) used a book-cover design task to examine the neural correlates of creative thinking^34^. The authors separately administered and compared generative and evaluative phases of designing book-covers and observed preferential recruitment of the DLPFC, ACC, and left fO-AI regions during the evaluative as compared to generative phase. Other neuroimaging studies, where participants were asked to generate a unique/unusual response to a given stimuli, have also suggested increased recruitment of similar task-control attentional networks during creative thinking^7^,^17^. In our fMRI task, we did not separate generate and evaluate phases of the task to keep the creative thought process as close to a real-world experience as possible. However, building upon previous studies, recruitment of the task-control regions during word-drawing suggests that even with no explicit instructions to produce novel solutions, participants were accentuating idea evaluation more than idea generation during our task.

To choose the most unique or unusual response, idea selection and evaluation is required and is evidently facilitated by task-control networks. It is, however, unclear how such preferential recruitment of task-control networks facilitates creativity and spontaneity during an improvisation. Interestingly, during a musical improvisation task, Limb and Braun found that enhanced creativity in expert musicians was associated with reduced recruitment of task-control networks^13^. Other, more recent studies, also done in expert musicians, show deactivations in the DLPFC during musical improvisation as a sign of reduced monitoring and volitional control^10^,^12^. In line with their findings, we also observed a negative relation between the beta-estimates from the cluster encompassing the left ACC/DLPFC regions and representation ratings on our fMRI task in a non-artist population, thereby providing suggestive evidence for a negative role of higher engagement of task-control regions during spontaneous improvisation.

The role of implicit processing, especially via cerebral-cerebellar connectivity, during creative thinking has been previously hypothesized^25^,^26^, based on the anatomical claim that the cerebellum can facilitate efficient manipulation of movements and mental representations alike^26^,^35^,^36^,^37^,^38^. Recent work by Pinho *et al.* bolsters this claim, by showing increased cerebral-cerebellar functional connectivity in expert musicians during improvisation^10^. In our fMRI task, participants would have required both manipulations of movements as well as mental representations to successfully draw the given word. Thus, one would expect the cerebellum to be progressively engaged with increasing representation as well as creativity ratings of each word. Interestingly, we found activation in the cerebellum to uniquely and linearly increase with increasing creativity ratings only and not with representation ratings.

The extant literature, mainly from the work in primates, points towards motor control and motor learning as a primary role for cerebral-cerebellar interactions^39^,^40^,^41^. However, recent research comparing topographical organization and origins of cerebral peduncle fibers in human and macaque brains provides support for the role of cerebral-cerebellar interactions in higher order cognitive function in humans. For example, Ramnani *et al.* (2006) showed that while macaque brains had a large proportion of cerebral peduncle fibers originating from the cortical motor system, human brains, on the other hand, had the largest contribution of cerebral peduncle fibers arising from the prefrontal cortex^42^. The prefrontal cortex has been associated with processing of more abstract information as compared to the cortical motor system^43^, suggesting that the human cerebellum is involved in neural functions beyond that associated with control of movement.

A theoretical analogue of control theory models that were used to explain the role of the cerebellum in motor control in cerebellum^25^,^39^ could also be employed to hypothesize how cerebral-cerebellar interaction might facilitate enhanced improvisation and creativity skills. To achieve speed, accuracy, and automaticity in motor command executions, researchers have proposed that the motor commands directed towards the movement control systems are also copied as “internal models” in the cerebellum. Accordingly, these internal models serve as cerebellar representations that can simulate natural body movements^44^. Through repeated and parallel simulations, the cerebellum facilitates acquisition of advanced motor skills and eventually provides automaticity. As proposed by others^25^,^26^,^39^,^43^, this theoretical model of motor control and learning can be extended to higher order cognitive functioning and thought processing. Along the same lines, we extend the putative role of the cerebellum to improvisation and creative thinking.

During our fMRI task, participants manipulate and amalgamate existing mental representations to express the given word in a sketch using the MR-safe tablet. We hypothesize that internal models of the cerebellum could facilitate such manipulations of mental representations, by simulating and parallelizing the sketching of the given word in multiple ways. Such simulations, would in turn, allow participants to more efficiently draw the target word. It remains unclear, however, how such greater efficiency is translated to creativity. Future research paradigms are required to systematically dissociate the cerebellum’s role in different aspects of creative thinking (e.g., elaboration, flexibility, fluency, originality, etc.). In sum, our results provide preliminary evidence of cerebellar activity associated with spontaneous improvisation and figural creativity and extend previous results to non-musicians/artists.

A potential limitation of our fMRI task arises from the possibility that zigzag-drawing might not fully account for the amount of language processing and/or overall cognitive load that is required during word-drawing. Thus, while contrasting these two conditions some activation could also be attributed to language processing and/or higher cognitive load. However, this potential limitation would not influence the results from the parametric analysis of the word-drawing condition. In the future, we plan to use control conditions that can better account for overall cognitive load (e.g., moving a pen through a maze, without touching boundaries) and language processing associated with the word-drawing condition.

We specifically developed our novel fMRI task using an efficient block design, with a total of 10 blocks per condition. However, the lack of significant correlation between subjective difficulty ratings and expert rating scores (both on creativity and representation scales) could be partially attributed to the small number of blocks per condition. Lastly, due to the nature of our experimental design and the fact that we could not record a timestamp for every stroke made by the participants, we cannot discern the neural resources employed purely during (pre-drawing) creative thinking versus implementation of the drawings. In the future, we can achieve such discernment by instructing participants to “imagine” or visually construct the representation before drawing/depicting one using the MR-safe tablet.

In sum, our results indicate a putative negative role of conscious monitoring and volitional control and a potentially positive role of implicit processing via cerebral-cerebellar interaction during spontaneous improvisation and figural creative thinking.

## Methods

### Participants and study design

Thirty-six healthy adults (18M, 18F) were initially enrolled in the study. Of these, one participant was excluded due to the use of prescription antidepressants; two participants were excluded due to excessive motion in the scanner, while three participants had incomplete data. Thus, the final data analyses were limited to 30 adult participants (16F, Mean Age = 28.77 years (S.D. = 5.54 years) and Mean IQ = 120 (S.D. = 10.53)). Participants were included in the study if they could undergo a magnetic resonance imagining (MRI) scan of the head and were right-handed. Participants were excluded if they self-reported a current or past history of psychiatric or neurological conditions that had lead them to consult a medical professional, or had metallic devices or implants in the head or body that are contraindicated for MRI. We recruited participants by sending out flyers via emails, message boards, list-servers, and word of mouth. Participants were recruited on or around the Stanford campus and surrounding areas. All experiments were performed in accordance with the relevant guidelines and regulations of Stanford University’s Institutional Review Board (Human Subjects Division), which approved all the experimental protocol and procedures. Written informed consent was obtained for every participant in the study.

### The fMRI Task

**Figure 1:**
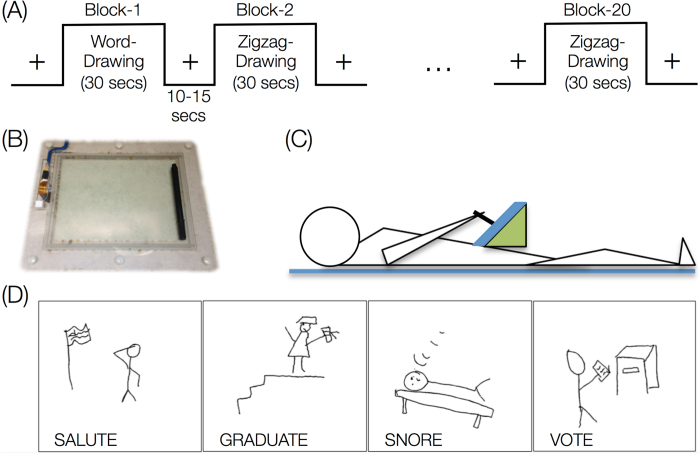
(A) Task was setup as a block design with two conditions (word-drawing and zigzag-drawing). (**B**) MR-safe tablet and pen. (**C**) Cartoon depicting how the participants used MR-safe tablet while lying down in the fMRI scanner. (**D**) Representative drawings from the word-drawing condition, drawn while participants were lying down in the fMRI scanner.

**Figure 2:**
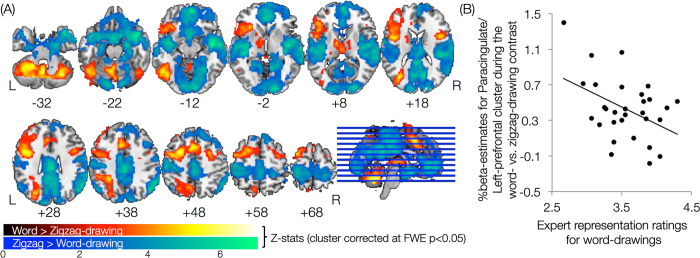
(A) Neural correlates of spontaneous improvisation and figural creativity. The red-yellow scale depicts contrast of word-drawing versus zigzag-drawing, while the blue-green scale represents the reverse contrast. (**B**) Correlations between beta-estimates from the word-drawing versus zigzag-drawing contrast and expert representation ratings.

**Figure 3:**
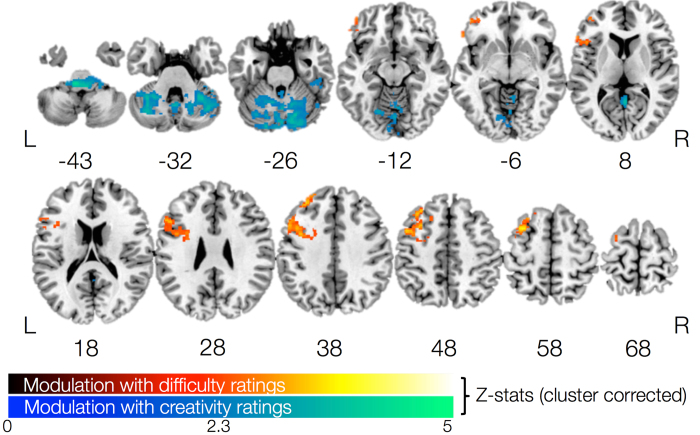
Parametric modulation of fMRI activation during word-drawing condition using self-reported difficulty ratings (in red-yellow color scale) and expert creativity ratings (in blue-green color scale) . No significant effect was found for the expert representation ratings.

The word-guessing fMRI task was based on the game of Pictionary^TM, ^45, and was developed using Matlab (http://www.mathworks.com) and Psychtoolbox version 3 (http://psychtoolbox.org) software. We used a block-design with 30 seconds block duration for each of the two conditions (word-drawing and zigzag-drawing). In the first condition, word-drawing, participants were asked to draw a given word (mainly actions or verbs) to the best of their ability using the MR-safe drawing tablet, with the caveat that others would later try to guess the word by their drawing alone. To control for the basic motor and visuospatial aspect during the word-drawing condition, participants were also asked to make a drawing representing the control word (“zigzag”) in the second condition. Each block was separated by a fixation period with a random duration within the range of 10–15 seconds (see Fig. 1). There were a total of 10 blocks per condition and the total duration of the task was approximately 14.5 minutes. In each condition, participants were shown a word on the top-left corner of the screen. Participants were asked to fully utilize the given 30 seconds in each block and continue to add elements to the illustration in case they wanted to finish early.

The words in the word-drawing condition were chosen from the pool of “action words” from the game of Pictionary^TM^. To balance the difficulty level in drawing different words, across participants, the chosen words were rated by a separate set of participants (N = 10) as “Difficult”, “Medium”, and “Easy” to draw. For example, drawing “cry” was rated as easy, while drawing “exhaust” was rated as difficult. Overall, we chose 3 difficult, 4 medium, and 3 easy words. The order of words was randomly chosen and was kept consistent across all participants. Additionally, participants in the fMRI study self-rated difficulty level (difficult, medium, or easy) for drawing each word during a post-scan questionnaire.

The MR-safe drawing tablet was designed and developed specifically for this study using an MR-compatible touch-sensitive surface. It uses a KEYTEC 4-wire resistive touch glass connected to a Teensy 2.0 with custom firmware. This device connected via USB port. It streamed the absolute position using a simple serial protocol. The tablet case was build out of clear acrylic using a laser cutter. The firmware for this tablet is made open-source and is available at https://github.com/cni/widgets/tree/master/touch.

### Behavioral Assessments

#### General intelligence

The Wechsler Abbreviated Scale of Intelligence-II (WASI-II) was used to measure general intelligence^46^. The WASI-II is designed to be administered individually in approximately 30 min. The measure consists of four subtests: Vocabulary, Similarities, Block Design, and Matrix Reasoning were used to obtain the Full Scale IQ (FSIQ). The WASI-II has a mean standard score of 100 with a standard deviation of 15.

#### Task performance

Two expert raters from the Stanford Design School (authors A.R. and G.H.) were chosen to blindly rate each drawing (from the word-drawing condition) on the scales of (a) representation and (b) creativity. Each drawing was de-identified and a web-based interface was developed for the raters for easy access to all illustrations. The instructions for the “representation” scale were as follows: “how easily do you think another person can guess the word represented by the drawing”. The ratings were obtained on a five-point scale (1-5), where 1 is “Not Representative”, 2 is “Little Representative”, 3 is “Moderately Representative”, 4 is “Representative”, and 5 is “Very Representative”.

The “creativity” rating of each drawing was assessed based on the three subscales of – fluency, elaboration, and originality. These subscales were chosen based on established standardized tests of figural creativity^47^. Scores from these three subscales were averaged to get the final score of creativity. Each subscale was defined as follows: (a) Fluency - total number of elements in the drawing; (b) Elaboration - imagination and exposition of detail; and (c) Originality - the statistical infrequency and unusualness/uniqueness of the response. The rating for each subscale was also done on a five-point scale (1-5).

Importantly, if any drawing was not clear (e.g., due to unintentional lines drawn by a multi-touch on the tablet), the raters marked those drawings as “confusing” and were not included in the analysis. Overall, less than 4% out of 300 drawings were excluded. The two raters were trained on a small sample of drawings (36 drawings) and their inter-rater reliability index for all the drawings (as measured by Intra Class Correlation Coefficient (ICC)) was 0.80 for representation and 0.884 for the creativity scale.

Lastly, as mentioned before, participants also self-rated the difficulty level for each drawing (as difficult, medium, or easy) in terms of difficulty in drawing during the post-scan questionnaire.

#### MRI image acquisition

Participants were imaged on a 3Tesla scanner (GE MR750, Milwaukee, WI) at the Stanford University’s Center for Cognitive and Neurobiological Imaging (CNI) using a 32-channel radiofrequency receive head coil (Nova Medical, Inc., Wilmington, MA). The participant’s head was stabilized by packing foam between the temples and the inner surface of the receiver coil to minimize motion during the scan, and a plethysmograph was placed on a finger on the left hand to monitor peripheral pulse. To restrict additional movement of hands, cushions were placed under the tablet and under participants’ arms. A total of 435 whole-brain volumes were collected on 42 axial-oblique slices (2.9 mm thick) prescribed parallel to the intercommissural (AC-PC) line, using a T2*-weighted gradient echo pulse sequence sensitive to blood oxygen level-dependence (BOLD) contrast with the following acquisition parameters: Echo Time (TE) = 30 ms, repetition time (TR) = 2000 msec, flip angle = 77°, FOV = 23.2 cm, acquisition matrix = 80 × 80, approximate voxel size = 2.9 × 2.9 × 2.9 mm. To reduce blurring and signal loss arising from field in-homogeneities, an automated high-order shimming method based on gradient echo acquisitions was used before acquisition of functional MRI scans. A high-resolution T1-weighted three-dimensional BRAVO pulse sequence acquisition was acquired for co-registration with the following parameters: Echo Time (TE) = 2.8 ms, repetition time (TR) = 7.2 ms, flip angle = 12°, FOV = 23 cm, slice thickness = 0.9 mm, 190 slices in the sagittal plane; matrix = 256 × 256; acquired resolution = 0.9 × 0.9 × 0.9 mm. The images were reconstructed as a 256 × 256 × 190 matrix.

#### fMRI Data Analysis

Functional MRI data processing was carried out using FEAT (FMRI Expert Analysis Tool) Version 6.00, part of FSL (FMRIB’s Software Library, www.fmrib.ox.ac.uk/fsl). The following pre-statisticsal processing steps were applied: motion correction using MCFLIRT^48^, non-brain removal using BET^49^, spatial smoothing using a Gaussian kernel of FWHM 5 mm, grand-mean intensity normalization of the entire 4D dataset by a single multiplicative factor, highpass temporal filtering (Gaussian-weighted least-squares straight line fitting, with sigma=50.0s), and probabilistic independent component analysis^49^,^50^ as implemented in MELODIC (Multivariate Exploratory Linear Decomposition into Independent Components) Version 3.10, part of FSL. After preprocessing, the functional data were registered to each individual’s high-resolution T1-weighted image, followed by registration to the MNI152 standard-space by affine linear registration using FMRIB’s Linear Image Registration Tool (FLIRT)^50^. For each participant, independent components were classified as “artifact” or non-artifact using an in-house semi-automatic artifact removal tool (SMART; similar to a tool made for the EEG data^51^). SMART uses the following rules to categorize each component as an artifact: (a) when the time series of a component is highly correlated (r >0.4) with motion profile only and not at all with the task design; or (b) when a component has most of its power (>70%) in the high frequency range. Once categorized, SMART produces an HTML based web-tool for quality check, where the operator can easily override SMART’s automatic classification. The quality check step was incorporated to make sure that the categorization of a component as an artifact was accomplished conservatively; e.g., if the time course of a component showed transient correlation with the task design, the components were retained as potentially containing BOLD signal. After this quality check, SMART uses fsl_regfilt utility (supplied with FSL) to regress out the artifactual components to recreate 4-D datasets to be used in generalized linear model analysis. Additionally, sharp motion peaks were detected using fsl_motion_outliers script (supplied with FSL) and were regressed out in addition to the six motion parameters (from MCFLIRT). Registration to high-resolution structural and standard space images was carried out using FLIRT. Time-series statistical analysis was carried out using FILM with local autocorrelation correction. Group-level analysis was carried out using FEAT (FMRI Expert Analysis Tool). Z (Gaussianised T/F) statistic images were thresholded using clusters determined by Z >2.3 and a (corrected) cluster significance threshold of P = 0.05^48^,^52^. Featquery tool (supplied by FSL) was used to extract percent change in parameter estimates for functionally defined (clusters of activations) regions of interests. MRIcron was used to visualize neuroimaging results on structural brain images. Talaraich Client was used to search for Brodmann areas for peak cluster activations. For the fMRI parametric modulation analysis, at the participant (*P*_i_) level, we subtracted the average value (across the 10 drawings made by participant *P*_i_) of expert creativity ratings from each creativity rating received by *P*_i_. Similarly, we subtracted the average value of subjective difficulty ratings from each of the 10 subjective difficulty rating provided by *P*_i_ for his/her drawings. This procedure of demeaning (or normalizing) was done before running the individual level multiple regression to reduce the effect of individual variance during the group-level analysis.

## Additional Information

**How to cite this article**: Saggar, M. *et al.* Pictionary-based fMRI paradigm to study the neural correlates of spontaneous improvisation and figural creativity. *Sci. Rep.* **5**, 10894; doi: 10.1038/srep10894 (2015).

## Figures and Tables

**Table 1:** Cluster statistics and locations for the (a-b) primary and reverse contrasts, and (c) parametric analysis.

**(A) Primary contrast: Word-drawing versus Zigzag-drawing**								
				**MNI Coordinates**				
**Cluster Index**	**Cluster size (number of voxels)**	**P-value**	**Z-max**	**X**	**Y**	**Z**	**Hemisphere**	**Brain Region**
6	9761	1.35E-17	6.22	−4	14	46	Bilateral	Paracingulate Gyrus
			6.02	−30	4	46	Left	Middle Frontal Gyrus
			5.92	−24	6	50	Left	Superior Frontal Gyrus
			5.63	−42	2	24	Left	Precentral Gyrus
			5.26	−38	−4	46	Left	Precentral Gyrus
			5.17	−42	−2	48	Left	Precentral Gyrus
5	5875	2.91E-12	6.15	0	−54	−28	Vermis	Cerebellum
			6.12	−34	−46	−36	Left VI	Cerebellum
			6.04	−10	−58	−26		Cerebellum
			5.88	34	−66	−32	R. Crus	Cerebellum
			5.82	0	−58	−32	Vermis	Cerebellum
			5.8	−2	−62	−30	Vermis	Cerebellum
4	3349	5.96E-08	5.34	−34	−78	22	Left	Lateral Occ. Cortex
			5.33	−34	−70	18	Left	Lateral Occ. Cortex
			5.17	−28	−48	40	Left	Superior Par. Lobule
			4.88	−26	−74	28	Left	Lateral Occ. Cortex
			4.85	−8	−68	52	Left	Precuneus
			4.82	−38	−82	14	Left	Lateral Occ. Cortex
3	1175	0.0015	4.53	−10	−8	6	Left	Thalamus
			4.27	−10	−4	2	Left	Thalamus
			4.23	0	−10	4	Left	Thalamus
			4.22	0	−22	6	Left	Thalamus
			4.19	−2	−1	4	Left	Thalamus
			3.64	12	−14	2	Right	Thalamus
2	801	0.0156	4.91	28	4	50	Right	Middle Frontal Gyrus
			4.09	30	−6	44	Right	Precentral Gyrus
			3.95	32	−2	58	Right	Middle Frontal Gyrus
			3.74	20	4	50	Right	Superior Frontal Gyrus
			3.24	18	12	56	Right	Superior Frontal Gyrus
			3.06	42	2	62	Right	Middle Frontal Gyrus
1	649	0.0442	5.29	44	6	22	Right	Precentral Gyrus
			2.46	46	26	8	Right	Inf. Frontal Gyrus
			2.4	40	22	14	Right	Inferior Frontal Gyrus
								
(B) Reverse contrast: Zigzag-drawing versus Word-drawing								
2	71900	0	7.65	46	−8	−8	Right	Central Oper. Cortex
			6.59	42	10	−22	Right	Temporal Pole
			6.51	−56	−14	2	Left	Planum Temporale
			6.48	4	54	−10	Bilateral	Frontomedial Cortex
			6.39	38	4	−22	Right	Temporal Pole
			6.37	−56	−18	4	Left	Planum Temporale
1	744	0.0229	5.08	−48	−66	42	Left	Lateral Occ. Cortex
			4.99	−54	−64	30	Left	Lateral Occ. Cortex
			4.55	−52	−64	38	Left	Lateral Occ. Cortex
			3.16	−42	−64	30	Left	Lateral Occ. Cortex
								
(C) Parametric fMRI Analysis								
*With subjective difficulty rating*								
1	2152	1.08E-05	3.91	−32	12	54	Left	Middle frontal gyrus
			3.88	−32	10	58	Left	Middle frontal gyrus
			3.74	−30	40	40	Left	Frontal pole
			3.65	−52	22	24	Left	Inferior frontal gyrus
			3.56	−32	2	34	Left	Precentral gyrus
			3.52	−46	6	46	Left	Middle frontal gyrus
								
*With expert creativity rating*								
1	4822	5.96E-08	4.65	32	−50	−36	Right	Cerebellum, Right VI
			4.36	20	−82	−28	Right	Cerebellum, Right Crus
			4.36	−8	−32	−44	—	Brain Stem
			3.88	6	−36	−44	—	Brain Stem
			3.85	20	−70	−30	Rights	Cerebellum, Right Cru
			3.82	−36	−48	−34	left	Cerebellum, Left VI
